# Research of Pathogenesis and Novel Therapeutics in Arthritis 2.0

**DOI:** 10.3390/ijms21218125

**Published:** 2020-10-30

**Authors:** Chih-Hsin Tang

**Affiliations:** 1 Department of Pharmacology, School of Medicine, China Medical University, Taichung 40001, Taiwan; chtang@mail.cmu.edu.tw; Tel.: +886-22052121 (ext. 7726); 2 Chinese Medicine Research Center, China Medical University, Taichung 40001, Taiwan; 3 Department of Biotechnology, College of Health Science, Asia University, Taichung 41354, Taiwan

**Keywords:** arthritis, treatment, molecular mechanisms, biomarkers, inflammatory cytokines, prevention

## Abstract

Arthritis has a high prevalence globally and includes over 100 types, the most common of which are rheumatoid arthritis, osteoarthritis, psoriatic arthritis, and inflammatory arthritis. All types of arthritis share common features of disease, including monocyte infiltration, inflammation, synovial swelling, pannus formation, stiffness in the joints and articular cartilage destruction. The exact etiology of arthritis remains unclear, and no cure exists as of yet. Anti-inflammatory drugs (NSAIDs and corticosteroids) are commonly used in the treatment of arthritis. However, these drugs are associated with significant side effects, such as gastric bleeding and an increased risk for heart attack and other cardiovascular problems. It is therefore crucial that we continue to research the pathogenesis of arthritis and seek to discover novel modes of therapy. This editorial summarizes and discusses the themes of the 27 articles published in our Special Issue “Research of Pathogenesis and Novel Therapeutics in Arthritis 2.0”, a continuation of our 2019 Special Issue “Research of Pathogenesis and Novel Therapeutics in Arthritis”. These Special Issues detail important novel research discoveries that contribute to our current understanding of arthritis.

Arthritis has a high prevalence globally and includes over 100 types, the most common of which are rheumatoid arthritis (RA), osteoarthritis (OA), psoriatic arthritis (PsA) and inflammatory arthritis. All types of arthritis share common features of disease, including monocyte infiltration, inflammation, synovial swelling, pannus formation, stiffness in the joints and articular cartilage destruction. The exact etiology of arthritis remains unclear, and no cure exists as of yet. Anti-inflammatory drugs (NSAIDs and corticosteroids) are commonly used in the treatment of arthritis. However, these drugs are associated with significant side effects, such as gastric bleeding and an increased risk for heart attack and other cardiovascular problems. It is therefore crucial that we continue to research the pathogenesis of arthritis and seek to discover novel modes of therapy.

Our call for papers for this Special Issue—Research of Pathogenesis and Novel Therapeutics in Arthritis 2.0—Attracted a diverse collection of articles, which were closely examined by a panel of expert reviewers, who selected 27 (18 research papers and 9 reviews) for publication in this Special Issue. The majority of the research papers investigated the pathogenesis of arthritis and new biomarkers; three examined novel strategies in the treatment of arthritis. All of these papers are discussed below.

(i) The pathogenesis of arthritis. Research performed by Kmiolek and colleagues offers insights into how the interplay between transcriptional factors and microRNAs (miRNAs) contributes to RA pathogenesis [1]. Sanz and colleagues describe early immunomodulation in DMARD-naïve patients with new-onset RA and different patterns of CD4^+^ modulation in methotrexate responders and nonresponders [2], while Lucchino and colleagues discuss their findings of early subclinical lung abnormalities in RA-associated autoimmunity, which may assist clinicians with the clinical management and therapeutic decisions in individual patients [3]. The preclinical research by Fujii and colleagues indicates that active exercise in established phase arthritis inhibits joint destruction and RA disease activity [4]. Upregulation of miR-941 in CD14^+^ monocytes enhances osteoclast activation in patients with PsA, report Lin and colleagues [5], while other investigations reveal that age-related differences exist in knee joint transcriptome during the development of post-traumatic OA (PTOA) in mice, which may contribute to a severe PTOA phenotype in older individuals [6]. Akagi and colleagues offer novel insights into the pathophysiological function of angiotensin II (Ang II) in bone erosion and systemic bone loss in arthritis and they have found that excessive systemic activation of the renin–angiotensin system (RAS) is a risk factor for progressive joint destruction, which has important implications for the clinical use of RAS inhibitors in RA [7]. Teixeira and colleagues have addressed the interplay between inflammation and bone injury in collagen-induced arthritis (CIA) animal models of RA and confirm that such models are appropriate for investigating the mechanisms of bone repair/regeneration in chronic inflammatory conditions [8].

(ii) New biomarkers. Khanfar and colleagues describe preclinical findings showing that the transcription factor Nkx2-3 appears to regulate the development of autoimmune arthritis, probably via B cell signaling [9]. Li and Zheng have identified novel targets of knee OA shared by synovium and cartilage, which may help with the development of novel OA therapies [10]. Belluzzi and colleagues describe inflammatory and fibrotic changes in OA infrapatellar fat pad (IFP) characteristics, which suggests that targeting fibrosis could effectively counteract the disease progression and associated pain in patients with OA [11]. Groma and colleagues discuss their findings of persistent human herpesvirus 7 (HHV-7) infection in 81.5% of their study subjects with OA and reactivation in 20.5% of the subjects [12]. The study researchers discuss the significance of the immune system reaction to a foreign antigen and the impact on inflammatory cells in OA [12]. Chang and colleagues describe findings suggesting that electronegative low-density lipoprotein (LDL) may promote atherogenesis by augmenting macrophage foam cell formation, upregulating CD11c expression, and enhancing the expression levels of atherosclerosis-related mediators in RA [13]. In another study, Hähnlein and colleagues discuss evidence suggesting that human lymph node stromal cells (LNSCs) from RA patients regulate peripheral tolerance, making these cells a novel target to exploit in tolerance maintenance and induction [14]. Work by Yu and colleagues suggests that targeting peptidylarginine deiminase 2 (PADI2) may be a novel strategy for controlling inflammation caused by macrophages [15].

(iii) Novel strategies in the treatment of arthritis. Chou and colleagues discuss how the inhibition of discoidin domain receptors 1 (Ddr1) reduces chondrocyte apoptosis and promotes chondrocyte autophagy, leading to the attenuation of cartilage degradation, and so may therefore be a potential disease-modifying therapy for preventing OA progression [16]. Mendez and colleagues describe how chronic antibiotic treatment prior to the initial injury reduces inflammation and slows the development of PTOA in mice [17]. Hirata and colleagues detail promising findings showing that taurine inhibits mitochondrial dysfunction associated with glucocorticoid administration, preventing osteonecrosis in rabbits and cultured osteocytes [18].

Reviews. The nine reviews discuss recent findings on novel pathogenic aspects of inflammatory joint diseases and interesting investigations into treatment aspects of arthritis. Ailioaie and Litscher discuss the benefits of using photobiomodulation in juvenile idiopathic arthritis and adult RA [19], while Krajewska-Włodarczyk and colleagues focus on the immunopathogenic and therapeutic role of microparticles in chronic immune-mediated inflammatory joint diseases [20]. Tsukazaki and Kaito discuss the role of the interleukin (IL-23/IL-17) pathway in the pathogenesis of spondyloarthritis (SpA) and clinical evidence for biological agents that target IL-23 and IL-17 in the treatment of SpA [21]. Hong and colleagues discuss biological and molecular mechanisms that contribute to arthritis pain in animal models of RA and OA [22], while Kotyla and colleagues detail the activity of Janus kinase (JAK) inhibitors in the cardiovascular system of patients with RA, as well as the side effects of these medications [23]. Mikhaylenko and colleagues summarize genetic polymorphisms associated with the development of RA and they discuss how to apply the genetic variants in the selection of anti-RA medications [24]. MacDonald and colleagues describe the immunopathogenic characteristics of melatonin in RA disease [25], while Tanikella and colleagues discuss emerging genome-editing techniques against chronic degenerative joint conditions such as OA [26]. Finally, Cici and colleagues review the role of Wnt signaling in RA and SpA, focusing on the effect of biological therapy on this pathway and its possible clinical implications [27].

We hope that this Special Issue will provide new research insights and directions that ultimately inspire new arthritis prevention and treatment strategies.
